# Case Report: An integrated proposal for nutritional periodization and body mass loss management in a world boxing council super-bantamweight champion

**DOI:** 10.3389/fspor.2026.1816128

**Published:** 2026-06-25

**Authors:** Carlos Abraham Herrera-Amante, Wiliam Carvajal-Veitía, Rodrigo Yáñez-Sepúlveda, José Francisco López-Gil

**Affiliations:** 1 Nutritional Assessment and Nutritional Care Laboratory, Division of Health Sciences, Tonalá University Center, University of Guadalajara, Tonalá, México; 2 Ibero-American Network of Researchers in Applied Anthropometry, Almería, Spain; 3 Subdirectorate of Teaching and Research, Institute of Sports Medicine, Havana, Cuba; 4 Faculty of Education and Humanities. School of Sports Science, Universidad Andres Bello, Viña del Mar, Chile; 5 Department of Sport Sciences, Faculty of Sport and Health Sciences, Fit Generation Research Institute, Andorra la Vella, Andorra; 6 School of Medicine, Universidad Espíritu Santo, Samborondón, Ecuador; 7 Vicerrectoría de Investigación y Postgrado, Universidad de Los Lagos, Osorno, Chile

**Keywords:** anthropometry, athletic performance, body composition, boxing, sports nutritional sciences

## Abstract

**Purpose:**

This case study presents a 25-week nutritional period and body mass management strategy for a Mexican world champion that competes in the super bantamweight category. While the primary goal was to achieve competition weight, the intervention revealed unexpected physiological adaptations to prolonged energy restriction.

**Methods:**

A structured nutritional plan was implemented using multiple indicators of nutritional status. Assessments included anthropometry, hemoglobin concentration, creatine kinase activity, and urine specific gravity. The resting metabolic rate (RMR) and respiratory exchange ratio (RER) were measured six times. Dietary intake was recorded via the ASA24® Dietary Assessment Tool. Strength was evaluated via isometric dynamometry (legs, chest, and back) and grip strength. VO_₂max_ was estimated pre- and postintervention via the Åstrand-Ryhming step test.

**Results:**

After 23 weeks, the athlete's body mass decreased by 23.7 kg (30.1%), and the sum of the six skinfolds decreased from 106 mm to 67 mm. The RER decreased from 0.92 to 0.78, indicating improved metabolic flexibility. VO_₂max_ increased from 42 to 58  mL/kg/min. The isometric strength increased from 120 kg to 150 kg, and the grip strength improved bilaterally. Notably, the RMR increased rather than decreased, and lean mass loss coexisted with improvements in physical function, challenging conventional expectations under energy deficit.

**Conclusion:**

These findings illustrate potential individual variability in physiological responses during prolonged energy restriction in weight-class sports. The coexistence of elevated RMR, changes in body composition, and improved physical function highlights the complexity of athlete adaptation and underscores the need for further research in elite combat sport athletes.

## 1 Introduction

In combat sports such as professional boxing, the ability to achieve and maintain optimal body composition is essential for both performance and competition (1, 2). Strict weight categories compel athletes to adopt rapid body mass loss strategies, which, although effective in the short term, are often associated with significant physiological stress, impaired recovery, and potential long-term health consequences (3). These challenges are particularly evident in lighter weight categories, where fat-free mass becomes the primary limiting factor for further body mass adjustments (2, 4).

Nutritional periodization has emerged as a practical approach to address these challenges by strategically aligning dietary intake with training, recovery, and competition phases (5–8). This method, which is widely applied in endurance sports, offers promising potential for optimizing performance while safeguarding athlete health (9–11). However, there is very limited evidence of its use in combat sports in the literature. The few available publications on combat often fail to describe such strategies in detail, leaving a significant gap in understanding their potential application in these disciplines (12–14).

This case study focuses on a Mexican professional boxer former WBC super-bantamweight world title 2013, exemplifying the unique challenges of integrating nutritional periodization into a high-performance context. This research aims to address the gap in the specialized literature by demonstrating how tailored dietary strategies can balance immediate competition demands with long-term athletic success, offering valuable insights for advancing nutritional practices in combat sports.

## 2 Methods

### 2.1 Athlete and case study background

The subject of this case study is a Mexican professional boxer (born February 11, 1983) and former WBC super-bantamweight champion. After losing his first title defense, he experienced reduced self-care and gained significant body mass. Three years later, aiming to regain a competitive form, he sought sports nutrition counseling to reach his division's competitive body mass. Over his career, he fought 47 bouts, securing 38 wins, 7 losses, and 2 draws.

At baseline, the athlete presented with a body mass substantially above his historical competitive range. This condition reflected a prolonged period of detraining, reduced structured physical activity, and suboptimal nutritional habits following retirement. Therefore, the initial body mass should not be interpreted as representative of his habitual competitive state, but rather as a detrained condition that contextualizes the magnitude of the subsequent body mass reduction.

While the CARE guidelines were considered during manuscript preparation, certain items (e.g., genetic information, detailed diagnostic reasoning, or differential diagnosis) were not included as they are not directly applicable to a performance-focused sports nutrition case without an underlying clinical condition. Nevertheless, additional contextual and medical background information has been incorporated to improve alignment with CAse REport (CARE) guidelines (15). The athlete reported no chronic medical conditions, no history of metabolic or cardiovascular disease, and no relevant family history. Psychosocially, he described reduced motivation and lifestyle instability during retirement, which contributed to weight gain. No previous structured nutritional or medical interventions had been implemented before the present program.

#### 2.1.1 Physical examination

A general physical examination was conducted at baseline. The athlete presented with central adiposity, normal resting blood pressure (118/72 mmHg), normal resting heart rate (62 bpm), and no musculoskeletal limitations. Postural assessment revealed mild thoracic kyphosis associated with detraining. No clinical signs of dehydration, anemia, or endocrine dysfunction were observed.

### 2.2 Timeline

**Figure 1:**
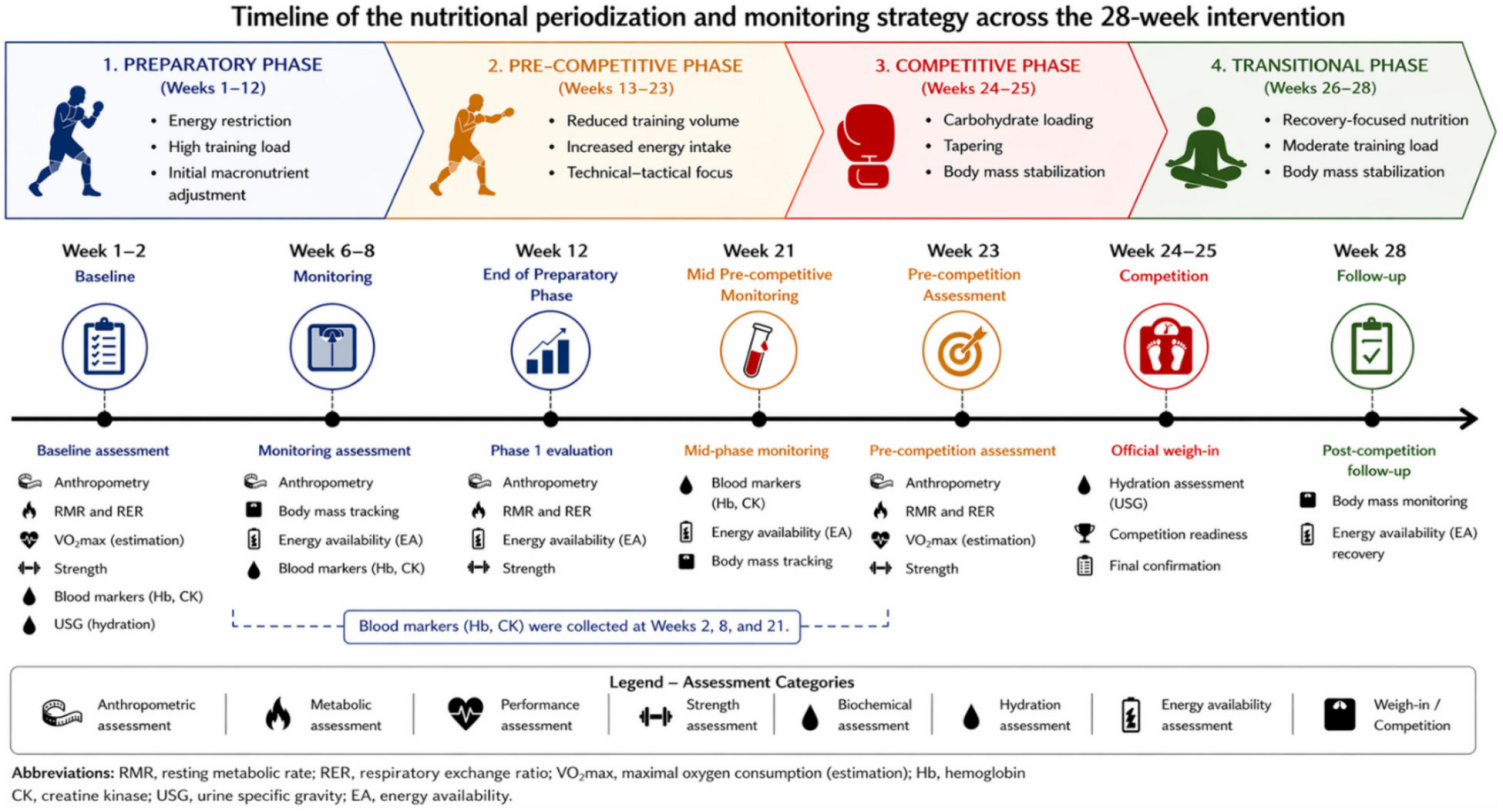
Timeline of the nutritional periodization and monitoring strategy across the 28-week intervention. The intervention was structured into four phases: preparatory (weeks 1–12), pre-competitive (weeks 13–23), competitive (weeks 24–25), and transitional (weeks 26–28). Assessments included anthropometry, resting metabolic rate (RMR), energy availability (EA), biochemical markers, and performance tests conducted at key time points throughout the intervention. Nutritional and training strategies were adjusted according to the specific objectives of each phase.

To contextualize the sequence of assessments and interventions across the 28-week program, a CARE-compliant timeline is presented below. This overview summarizes the key clinical, nutritional, and performance-related events before the detailed description of the integrated nutritional periodization strategy Figure 1.

### 2.3 Anthropometric assessment

Anthropometric assessments were conducted following ISAK standards (16), by a Level 3 certified anthropometrist. The instruments used included a SECA® 874 digital scale for body mass, a SECA® 217 stadiometer for stretch stature, a Slim Guide® skinfold caliper, a SmartMet® flexible metal tape for girths, and a SmartMet® bone caliper for breadths.

All instruments were calibrated prior to each measurement session according to the manufacturer's specifications. The Slim Guide® caliper was checked via calibration blocks, and the digital scale was verified with certified weights. The stadiometer was recalibrated weekly to ensure vertical alignment.

The technical error of measurement (TEM) was calculated for skinfolds and girths via duplicate measurements on 10 subjects prior to the study, yielding intraevaluator TEMs of 2.1% for skinfolds and 1.3% for girths, within acceptable ISAK thresholds.

Although DXA-based equations offer high precision, the use of ISAK anthropometry was chosen for its practicality, reproducibility, and accessibility in field settings. The Jackson and Pollock equation (17) was used to estimate the body fat percentage and fat-free mass (FFM). Its high correlation in previous studies and low standard error make it a reliable tool for longitudinal monitoring throughout different phases of the intervention. A multiethnic validation study reported acceptable fit indicators in Hispanic males (R = 0.91; SEE = 3.0%), supporting its applicability in the present case study involving a Mexican athlete (18).

### 2.4 Resting metabolic rate (RMR)

RMR was measured via indirect calorimetry (KORR® Cardiocoach Plus - model 9002-CO2, Germany). The system was calibrated before each use via a certified gas mixture and an ambient air calibration protocol. The coefficient of variation (CV) for repeated measures was <5%, and the device's manufacturer-reported accuracy was ±3%.

Before each measurement, the participant underwent a 5– to 10-minute training session to familiarize themselves with the equipment. This session included breathing practice with the mask, an explanation of the measurement protocol, and a brief simulation to reduce anxiety and ensure steady-state conditions. The RMR assessment was conducted while the participant remained in a supine position for an average of 15 min. Following Compher et al. (19), the first 5 min were discarded, and the average of the remaining 10 min was used for analysis, ensuring a steady-state respiratory exchange ratio (RER) and oxygen consumption.

### 2.5 Energy availability (EA)

EA was estimated using the following equation (20):

$$\begin{array}{rl} & \mathrm{EA}\;(\mathrm{kcal}\cdot \mathrm{kg}\;\mathrm{FF}M^{-1}\cdot \mathrm{da}y^{-1})\\ & =\frac{\mathrm{Energy}\;\mathrm{Intake}\;(\mathrm{kcal})-\mathrm{Exercise}\;\mathrm{Energy}\;\mathrm{Expenditure}\;(\mathrm{Kcal})}{\mathrm{Fat}-\mathrm{Free}\;\mathrm{Mass}\;(\mathrm{kg})} \end{array}$$

Fat-free mass (FFM) was estimated from the body fat percentage obtained via the Jackson and Pollock equation (17, 18).

Energy intake was assessed through a structured 3-day dietary recall interview conducted by a certified nutritionist, supported by the ASA24® Dietary Assessment Tool. Portion sizes were verified via food models and photographic guides.

Exercise energy expenditure (EEE) was estimated via Model 2 from Hiilloskorpi et al. (21) on the basis of weekly average heart rate monitoring with a Polar FT5 sensor. EEE was calculated as net expenditure (excluding RMR during exercise) to improve EA accuracy, as recommended by Areta et al. (22).

### 2.6 Maximal oxygen consumption assessment

The maximal oxygen consumption (VO_₂max_) was estimated via the Åstrand and Ryhming (23), step test, where the subject stepped up and down a 40 cm step for 5 min at 22.5 cycles per minute, regulated by a metronome set to 90 bpm. Heart rate was recorded at the end of the fifth minute to estimate VO_₂max_.

The test was conducted under standardized conditions (ambient temperature 20–22 °C, morning hours, postrest), and heart rate was measured via a Polar FT5 monitor. The Åstrand nomogram was used to estimate VO_₂max_, adjusted for age and sex.

### 2.7 Isometric strength

Isometric strength in the leg, chest, and back, as well as grip strength in both arms, was measured via T16 K and SMEDLEY III T-16 K isometric dynamometers (Takei Scientific Instruments, Shinagawa-Ku, Tokyo).

Measurements were taken in triplicate, with the highest value recorded. Calibration of the dynamometers was performed monthly according to the manufacturer's protocols. Standardized positioning and verbal encouragement were used to ensure maximal effort.

### 2.8 Urine specific gravity (USG)

The USG was measured at different times during preparation via a digital refractometer (ATAGO® PAL-10S). The refractometer was calibrated with distilled water before each use. Measurements were taken from midstream urine samples collected in the morning, and values were interpreted on the basis of the criteria of Armstrong et al. (24), for hydration status.

### 2.9 Ethical considerations

The athlete has read, approved, and provided written consent for the publication of this study, which adheres to the ethical standards established for case studies. All assessments were conducted following the ethical principles of the Declaration of Helsinki for human studies (25).

### 2.10 Statistical analysis

All statistical analyses were performed using RStudio (version 2024.04.2 + 764, Posit Software, PBC, Boston, MA, USA).

## 3 Results

### 3.1 Integrated nutritional periodization strategy

**Figure 2:**
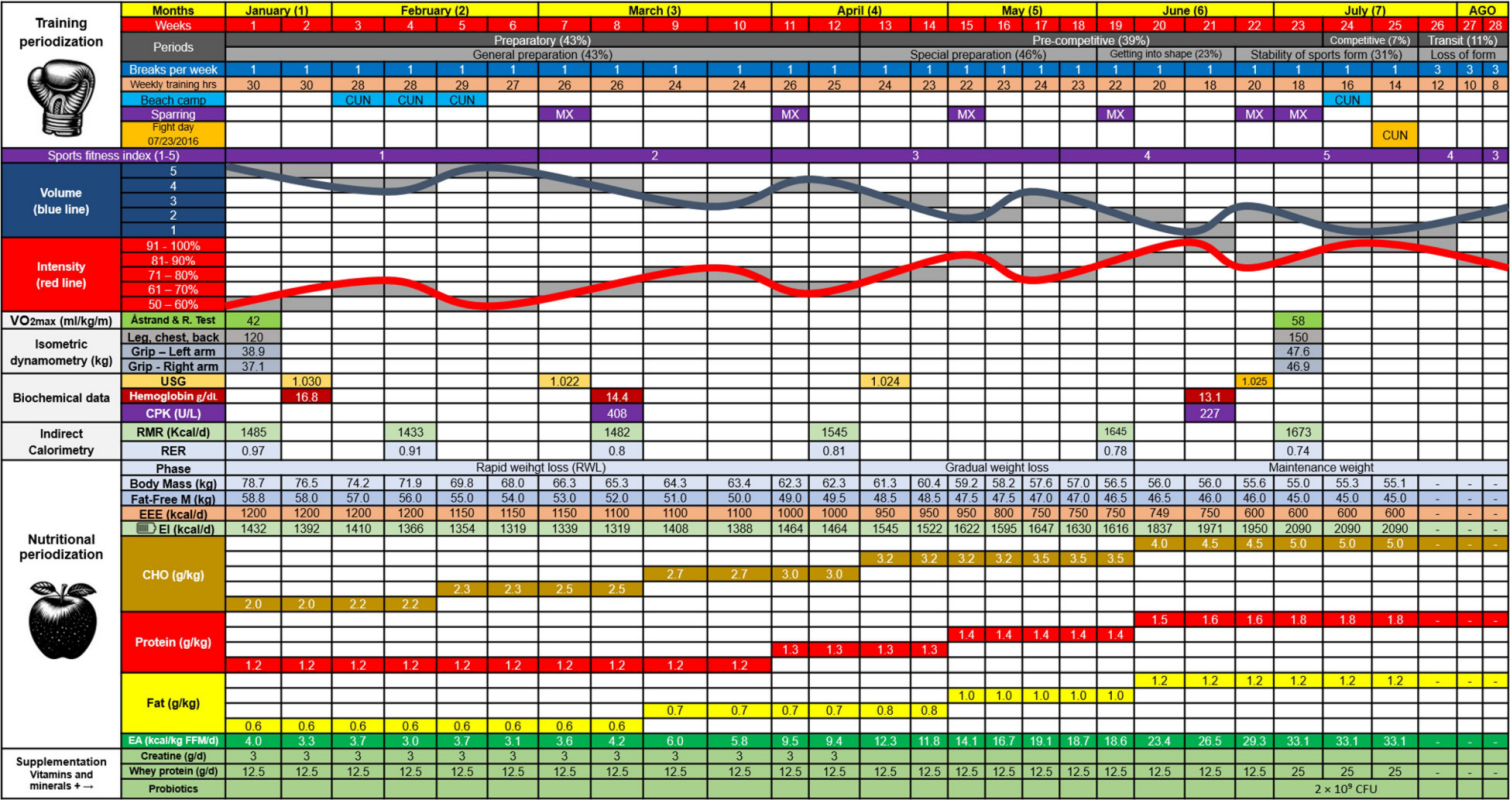
Periodization table displaying an athlete's training, nutrition, competition, and physiological parameters over twenty-eight weeks, including phases for preparation, competition, and transition, weekly training loads, volume and intensity lines, body composition metrics, biochemical data, calorimetry results, macronutrient intake, and supplementation schedules.

The integrated nutritional periodization strategy, detailed in Figure 2, was structured across four phases to optimize body mass management, energy availability (EA), and performance while monitoring biochemical and functional adaptations. The characterization of the phases is as follows:

#### 3.1.1 Preparatory phase (weeks 1–12)

- Body mass decreased from 78.7 kg to 68.0 kg (−10.7 kg).
- EEE decreased from 1,200 kcal/day to 1,000 kcal/day, whereas EI ranged from 1,432 to 1,464 kcal/day.
- EA increased from 4.0 to 9.4 kcal/kg FFM/day.

Despite the reduction in body mass from 78.7 kg to 68 kg during the first six weeks, fat-free mass (FFM) also declined notably, from 58.8 kg to 54 kg, indicating that the initial weight loss was not exclusively due to fat mass reduction. This loss was accompanied by significant decreases in skinfold thickness, suggesting a combined depletion of both adipose and lean tissue. The RMR fluctuated between 1,485 and 1,520 kcal/day during this phase, reflecting early metabolic adaptations to the energy deficit and changes in body composition.

#### 3.1.2 Pre-competitive phase (weeks 13–23)

- Body mass decreased from 62.3 kg to 58.0 kg (−4.3 kg).
- EEE declined to 749 kcal/day, and EI peaked at 1,937 kcal/day.
- EA increased to 23.4 kcal/kg FFM/day.

The reduction in EEE was due to a shift in training structure, with increased technical and tactical sessions replacing high-volume conditioning. Heart rate data confirmed a lower average exertion. The RMR unexpectedly increased to 1,637 kcal/day by week 23, despite a reported decrease in FFM. This discrepancy may reflect improved metabolic efficiency or measurement variability and warrants further investigation.

#### 3.1.3 Competitive phase (weeks 24–25)

- Body mass stabilized between 55.4 kg and 55.1 kg.
- EA reached 33.1 kcal/kg FFM/day, remaining above the recommended threshold.

This phase included carbohydrate loading and a reduced training volume, contributing to improved EA and recovery. The participant maintained their hydration status (USG <1.020) and reported high subjective readiness scores.

#### 3.1.4 Transitional phase (weeks 26–28)

- The training intensity decreased to 81%–90%, and the volume increased moderately.
- Slight body mass gain was anticipated.

EA was maintained at ∼28 kcal/kg FFM/day, which is slightly below the optimal value but sufficient for recovery. No significant changes in biomarkers were observed.

### 3.2 Energy intake, macronutrient distribution, and supplementation

- The carbohydrate content increased progressively, peaking at 6 g/kg during the competitive phase.
- Protein intake peaked at 1.8 g/kg, especially during recovery mesocycles.
- Lipid intake reached 1.2 g/kg in the moderate-intensity phase.

The supplements included creatine (3 g/day) during the preparatory phase (discontinued in week 12), whey protein postexercise, multivitamins, minerals, and probiotics during the final 4 weeks to support immune and gastrointestinal health. All supplements were tested by a third party to ensure compliance with anti-doping regulations.

### 3.3 Biomarkers and adaptations to training

- Hemoglobin decreased from 16.8 g/dL to 13.1 g/dL.
- The CPK levels decreased from 408 U/L to 227 U/L.
- The RMR increased from 1,485 kcal/day to 1,637 kcal/day.
- VO_₂max_ improved from 42 to 58 mL/kg/min.

The increase in VO_₂max_ was consistent with improved cardiovascular conditioning and reduced body mass. Isometric strength improved: leg, chest, and back strength increased from 120 kg to 150 kg; handgrip strength increased from 38.9 kg to 47.6 kg (left) and from 37.1 kg to 46.9 kg (right).

### 3.4 Changes in the anthropometric profile

- The thickness of the triceps and supraspinal skinfolds decreased by approximately 30% in the first 12 weeks (Table 1).
- Reductions >20% were noted in the iliac crest, subscapular, and abdominal regions.

**Table 1:** Anthropometric profile and changes in chronic phase.

Variable	Variable	Week 1	Week 12	Week 23	*Δ*1→2 (%)	*Δ*2→3 (%)	*Δ*1→3 (%)
Basics	Body mass (kg)	78.7	62.3	55.0	−20.8	−11.7	−30.1
	Stretch stature (cm)	167.2	167.2	167.2	0.0	0.0	0.0
Skinfolds (mm)	Triceps	13.0	9.0	8.0	−30.8	−11.1	−38.5
	Subscapular	18.0	14.0	12.0	−22.2	−14.3	−33.3
	Biceps	5.0	4.0	3.5	−20.0	−12.5	−30.0
	Pectoral	12.0	10.0	9.0	−16.7	−10.0	−25.0
	Mid-axillary	12.0	11.5	10.0	−4.2	−13.0	−16.7
	Iliac crest	17.0	13.5	12.0	−20.6	−11.1	−29.4
	Supraspinal	18.0	12.0	8.0	−33.3	−33.3	−55.6
	Abdominal	22.0	17.0	16.0	−22.7	−5.9	−27.3
	Front thigh	21.0	17.0	14.0	−19.0	−17.6	−33.3
	Medial calf	14.0	10.0	8.0	−28.6	−20.0	−42.9
Girths (cm)	Arm (relaxed)	31.5	30.7	30.2	−2.5	−1.6	−4.1
	Arm (flexed and tensed)	32.2	32.7	33.1	1.6	1.2	2.8
	Waist (minimum)	85.7	83.0	79.2	−3.2	−4.6	−7.6
	Gluteal (hips)	98.5	97.0	95.0	−1.5	−2.1	−3.6
	Thigh (mid-trochtib.lat.)	53.4	53.0	51.0	−0.7	−3.8	−4.5
	Calf (maximum)	35.5	35.3	35.1	−0.6	−0.6	−1.1
Breadths (cm)	Humerus	7.6	7.6	7.6	0.0	0.0	0.0
	Bistyloid	6.1	6.1	6.1	0.0	0.0	0.0
	Femur	11.0	11.0	11.0	0.0	0.0	0.0
*∑* Skinfolds	*∑*6 SF^a^	106.0	79.0	66.0	−25.5	−16.5	−37.7
	*∑*8 SF^b^	152.0	118.0	100.5	−22.4	−14.8	−33.9
Somatotype	Endomorphy	5.0	3.7	2.9	−27.0	−20.7	−42.1
	Mesomorphy	7.0	7.2	7.3	2.9	1.3	4.2
	Ectomorphy	0.5	2.3	3.6	406.9	56.1	691.2
	X	−4.5	−1.3	0.7	−71.0	−156.9	−116.5
	Y	8.5	8.4	8.0	−1.4	−4.4	−5.8
	SAD	0.0	2.3	3.8	-	65.1	-
Indexes	BMI (kg/m^2^)	28.15	22.29	19.67	−20.8	−11.7	−30.1
	AKS	1.4	1.1	1.0	−16.3	−8.7	−23.6

AKS, Aktiven Körpersubstanz index: BMI, Body mass index; ∑6SKF, Sum of 6 skinfolds; ∑8SKF, Sum of 8 skinfolds.

a Sum of triceps, subscapular, supraspinal, abdominal, front thigh, and medial calf skinfold thicknesses.

b Sum of triceps, subscapular, biceps, iliac crest, supraspinal, abdominal, front thigh, and medial calf skinfold thicknesses.

The anthropometric somatotype and its measure of variability (Somatotype Attitudinal Distance, SAD) were obtained using the Heath-Carter methodology (28).

**Figure 3:**
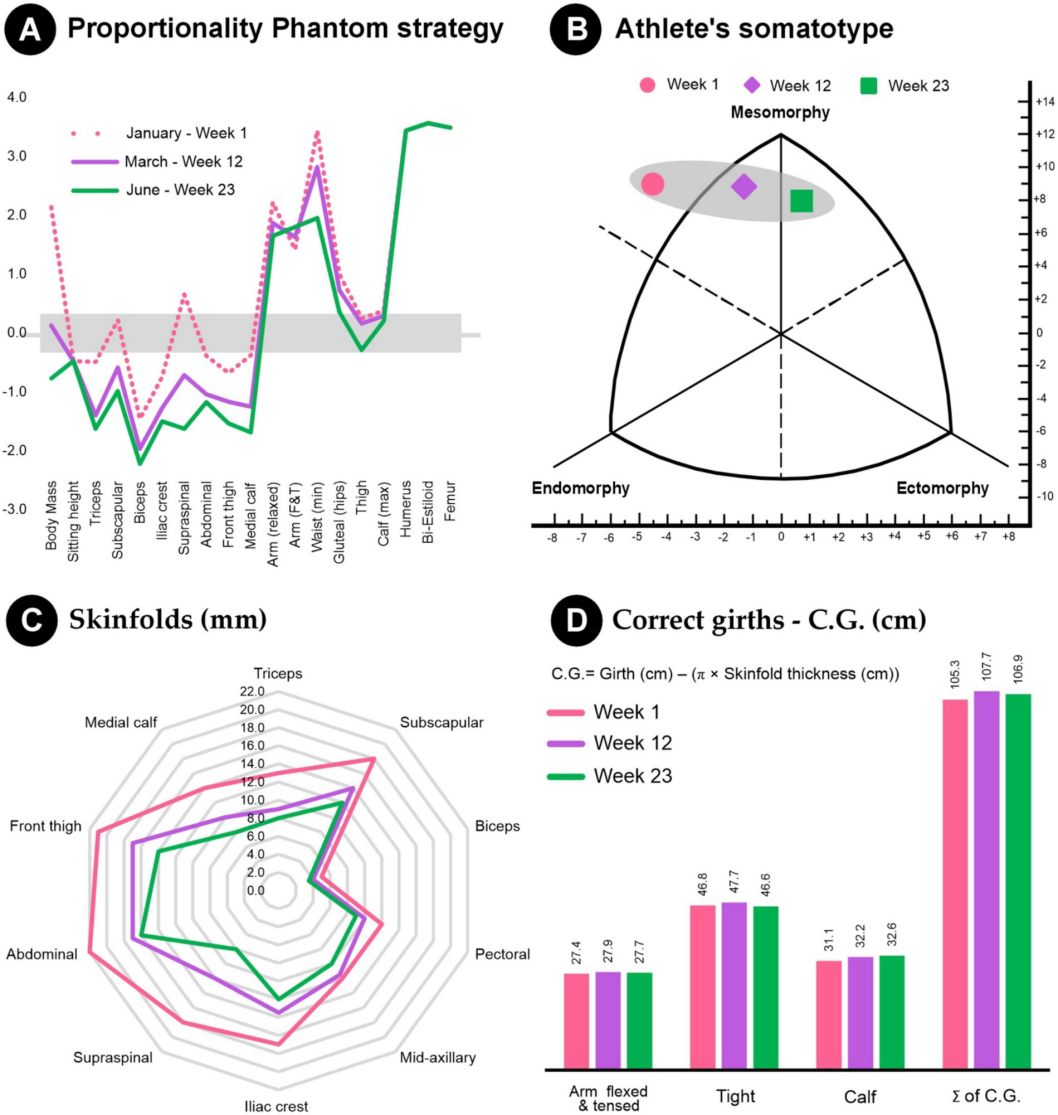
Four-panel graphic presenting the athlete's anthropometric changes over 23 weeks. Panel A displays the athlete's anthropometric profile expressed as Phantom Z-scores for selected dimensions at weeks 1, 12, and 23, illustrating changes in proportionality relative to the Phantom strategy. Panel B presents somatotype positions on a somatochart, showing a temporal shift characterized by reduced endomorphy and increased ectomorphy, with mesomorphy remaining the predominant component. Panel C is a radar chart illustrating reductions in skinfold thickness across multiple anatomical sites over the three assessment periods. Panel D is a bar graph of corrected girths, showing increases in arm, thigh, and calf corrected girths toward week 23.

The sum of the skinfolds decreased from 112 mm to 68 mm, indicating substantial fat loss. The somatotype shifted from “endomorphic Mesomorph” to “ectomorphic Mesomorph” over 23 weeks. The corrected girths remained stable, suggesting preservation of lean mass in key regions. Figure 3 illustrates proportional changes across the evaluated variables: (A) shows the proportionality graph highlighting reductions in subscapular, supraspinal, and abdominal skinfolds; (B) displays the somatochart transition from “Endomorphic Mesomorph” to “Ectomorphic Mesomorph”; (C) presents a radar graph of regional fat loss; and (D) depicts the stability of corrected girths throughout the intervention.

## 4 Discussion

This case study examined a 25-week nutritional intervention in a former WBC Super-Bantamweight world champion returning to elite competition after three years of retirement. In contrast to the rapid weight-cutting practices frequently described in combat sports (1–3, 13, 26), the strategy implemented here prioritized gradual body mass reduction within a structured framework of nutritional periodization (5). The objective was to progressively reduce fat mass while maintaining the evaluated physical capacities and limiting the physiological strain commonly associated with acute weight manipulation (4, 12, 14). Importantly, caloric intake was deliberately maintained above RMR throughout the intervention, avoiding prescriptions below this threshold.

A central finding was the marked reduction in estimated fat-free mass of 13.8 kg. In isolation, a decrease of this magnitude could suggest relevant structural tissue loss, particularly in contexts of sustained energy restriction. The IOC consensus statement on Relative Energy Deficiency in Sport describes endocrine and metabolic alterations when low energy availability (LEA) is prolonged (19). Experimental evidence indicates that EA below approximately 30 kcal per kg of fat-free mass per day may impair myofibrillar protein synthesis and alter endocrine signaling even in trained individuals (21). Although full endocrine profiling was not performed in this case, these frameworks provide a reference for interpreting the observed changes in body composition.

Despite the reduction in estimated fat-free mass, the evaluated physical capacities did not deteriorate. Both cardiorespiratory function and isometric strength improved during the preparation period. However, it is important to acknowledge that the Åstrand–Ryhming step test provides an indirect estimation of VO_₂max_. This method is sensitive to changes in body mass, heart rate variability, and the assumptions embedded in the nomogram. Consequently, part of the observed increase in estimated VO_₂max_ may reflect mathematical or methodological influences rather than a purely physiological enhancement. This limitation should be considered when interpreting the magnitude of the improvement. Research in combat sports shows that performance-related variables may remain stable when body mass reduction is gradual and strategically managed (1). Nutritional periodization models also indicate that training adaptations can be preserved during controlled phases of energy manipulation (5, 9, 26). In addition, Trexler et al. (2014) proposed that under energy-constrained conditions the organism may prioritize locomotor and aerobic function over anabolic processes. This perspective may help explain why functional expression improved even as estimated structural mass declined.

The anthropometric profile provides further context. Although fat-free mass derived from the Jackson and Pollock (1985) quadratic regression equation progressively decreased, corrected girths of the arm (flexed and contracted), thigh, and calf, as well as their sum, remained relatively stable over 23 weeks. Corrected girths were calculated by subtracting π multiplied by the corresponding skinfold thickness in centimeters from the measured girth at the same anatomical site, following classical anthropometric principles (27), and ISAK procedures (16). In elite combat athletes, these variables have shown sensitivity for monitoring peripheral muscularity over time (29, 30).

Given the reliance on field-based anthropometric equations, the magnitude of the estimated fat-free mass reduction must be interpreted cautiously. These models are sensitive to hydration status, tissue redistribution, and non-linear mathematical behavior when skinfold sums decrease substantially. This may partially explain the discrepancy between estimated fat-free mass loss and the relative stability of corrected girths.

In contrast, consistent longitudinal reductions were observed in skinfold thicknesses, reflecting decreased subcutaneous adiposity. The divergence between predictive fat-free mass estimates and the relative stability of corrected girths may partly reflect the mathematical behavior of the Jackson and Pollock model. Because it is based on quadratic regression, reductions in body mass and skinfold sums can induce non-linear shifts in estimated body density and consequently in calculated fat-free mass. Field-based equations are also sensitive to hydration status and tissue redistribution (31). These factors must be considered when interpreting large changes in estimated fat-free mass in longitudinal monitoring.

The nutritional structure of the intervention is relevant. At no point did prescribed caloric intake fall below measured resting metabolic rate. Although total EA was not calculated including non-exercise activity thermogenesis or the thermic effect of food, maintaining intake above RMR represented a conservative strategy intended to reduce the likelihood of severe energy suppression. Trexler et al. (32), described RMR suppression as a typical adaptation to sustained LEA, while Areta et al. (21), and Jeukendrup, et al. (33), noted that the relationship between EA and metabolic rate is not linear and may vary by context. In this case, the absence of a clear metabolic decline suggests that the applied strategy may have mitigated more pronounced adaptive downregulation, although this interpretation must remain cautious.

The unexpected increase in RMR should not be interpreted as evidence contradicting existing RED-S or LEA frameworks. Instead, it highlights potential individual variability in metabolic responses during prolonged energy restriction. Without endocrine profiling, no definitive conclusions can be drawn regarding the underlying mechanisms.

Biochemical monitoring provided complementary information. Hydration status was assessed through urine specific gravity following established recommendations (23), minimizing the probability that acute fluid shifts substantially distorted anthropometric estimations. Hemoglobin concentration decreased from 16.8 g per dL at week 2 to 14.4 g per dL at week 8 and 13.1 g per dL at week 21. Although this downward trend warrants attention, values remained within clinical reference ranges for adult males. Iron deficiency anemia is frequently reported in athletes exposed to high training loads and dietary manipulation. The absence of overt anemia is consistent with the preserved and improved aerobic capacity observed in this case.

Creatine phosphokinase was incorporated as an internal marker to assist with load regulation. The value of 408 U per L at week 8 coincided with a period characterized by very high training volume, including up to three daily sessions. By week 21, CPK had decreased to 227 U per L, aligning with a reduction in training volume during tapering. This pattern reflects coordinated adjustments between nutritional intake and workload management rather than isolated dietary manipulation.

Several limitations must be acknowledged. This is a single-athlete case, which limits external validity. Endocrine biomarkers such as testosterone, IGF-1, leptin, and thyroid hormones were not assessed. Angelidi et al. (34), emphasized that endocrine responses to LEA can vary considerably between individuals and may present without overt clinical signs. Therefore, subclinical adaptations cannot be excluded. Body composition was estimated through anthropometry rather than reference methods such as DXA, although procedures followed international standards (16). EA calculations did not incorporate all components of daily energy expenditure. These elements influence the precision of interpretation.

The findings reflect the context of an elite male boxer with extensive competitive experience and multidisciplinary supervision. Training history, psychological readiness, and structured support likely influenced the observed adaptations. Extrapolation to other athletes or competitive levels should therefore be approached with caution.

This case indicates that a prolonged and structured reduction in body mass, implemented without caloric intake falling below RMR and coordinated with training load management, was associated with substantial reductions in subcutaneous adiposity, relative stability of corrected muscular girths, improvements in evaluated physical capacities, and controlled biochemical markers of hydration and muscular stress. The marked decline in estimated fat-free mass highlights the need to interpret predictive equations within a broader monitoring framework when working with elite athletes in weight-class sports.

## 5 Conclusions

This case illustrates the complex and individualized physiological responses that may occur during prolonged nutritional periodization in a former elite boxer returning to competition. Improvements in aerobic and neuromuscular performance, together with the absence of metabolic suppression, suggest that carefully structured strategies may support functional adaptations even under constrained energy availability. However, these findings derive from a single athlete and should not be generalized. Future research involving larger samples and comprehensive endocrine monitoring is needed to refine current models of athlete monitoring in weight-class sports.

## Patient perspective

The athlete reported initial difficulties adapting to the energy restriction phase, including perceived fatigue and dietary adherence challenges. However, progressive improvements in perceived energy levels, training capacity, and overall well-being were noted during subsequent phases, particularly following adjustments in energy intake and training load. The athlete reported high perceived readiness during the competitive phase and expressed overall satisfaction with the intervention outcomes.

## Data Availability

The original contributions presented in the study are included in the article, further inquiries can be directed to the corresponding author.
